# Supplementary material for: Manual Therapy Improves Fibromyalgia Symptoms by Downregulating SIK1
Source: Int J Mol Sci. 2024 Sep 1;25(17):9523. doi: 10.3390/ijms25179523 (PMC11394909; doi:10.3390/ijms25179523)
Supplement: Supplementary file 1 [file ijms-25-09523-s001.zip › Supplementary Figure S1.pdf]

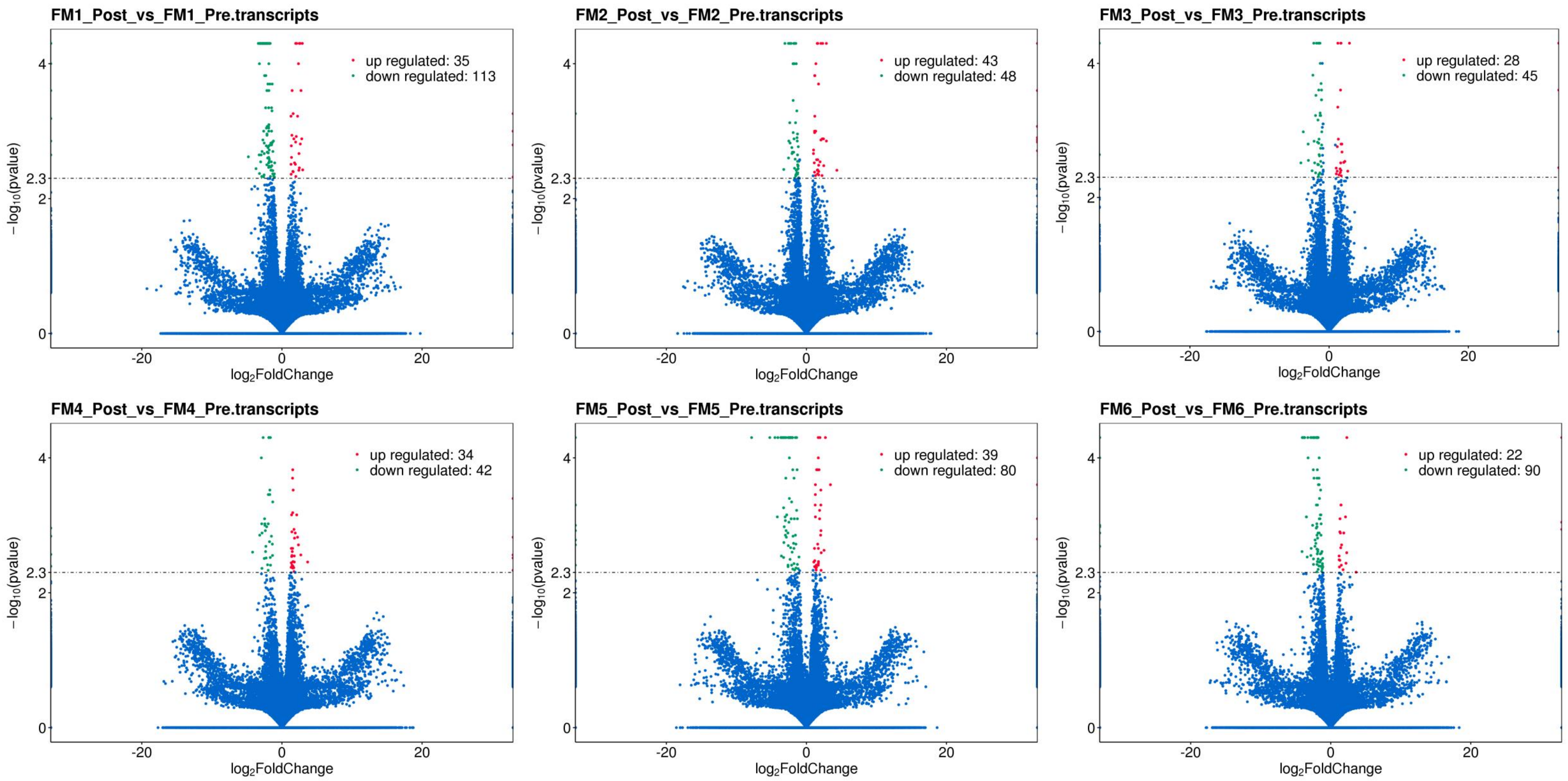

Supplementary Figure S1. Volcano plot representation of differential gene expression in PBMCs of FM with therapy by FM participant (FM1 through FM6, as indicated). Log2FoldChange values (X axis) are displayed with respect to -log10 of their p-values (Y axis), significance set at p<0.05 (FDR<0.1).
