# Supplementary material for: Manual Therapy Improves Fibromyalgia Symptoms by Downregulating SIK1
Source: Int J Mol Sci. 2024 Sep 1;25(17):9523. doi: 10.3390/ijms25179523 (PMC11394909; doi:10.3390/ijms25179523)
Supplement: Supplementary file 1 [file ijms-25-09523-s001.zip › Supplementary Figure S2.pdf]

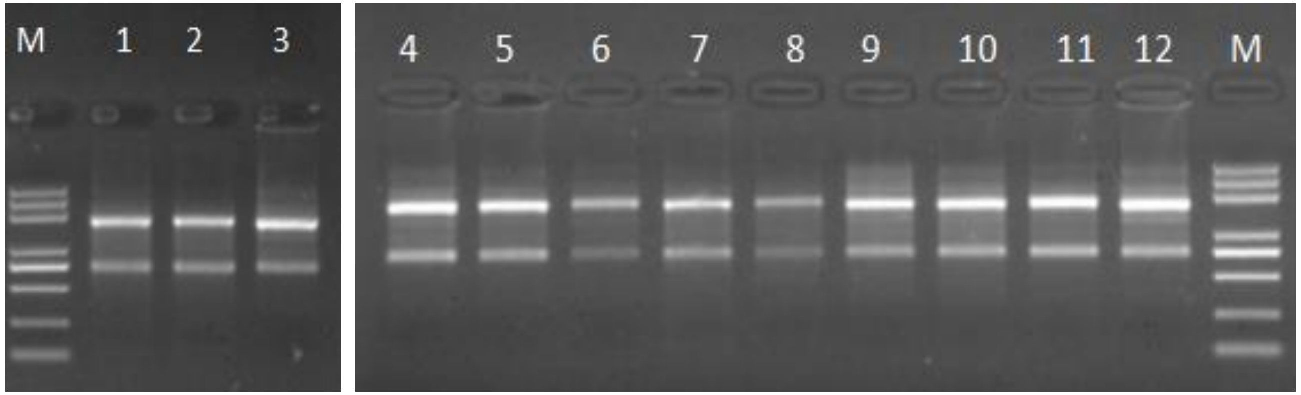

Supplementary Figure S2. QC analysis of total RNA by agarose gel electrophoresis (M: Trans 2K Plus Ladder, 100bp; 250bp; 500bp; **750bp**; 1kb; 2kb; 3kb and 5 kb) and RIN (RNA Integrity Number) analysis (2100 Bioanalyzer, Agilent) (individual electropherograms for samples FM 1 through FM 12 are shown below).

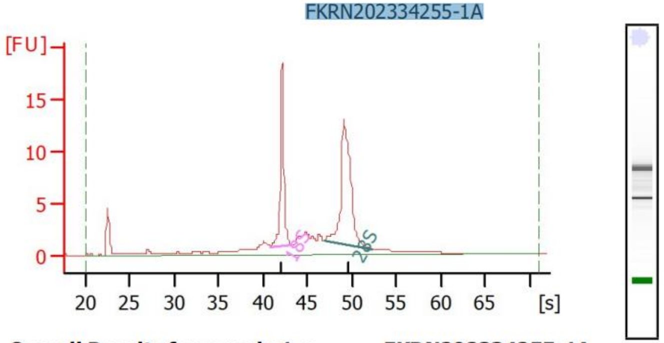

Overall Results for sample 1 : FKRN202334255-1A

RNA Area:	95.4
RNA Concentration:	179 ng/µl
rRNA Ratio [28s / 18s]:	1.7
RNA Integrity Number (RIN):	9.2 (B.02.10)
Result Flagging Color:	
Result Flagging Label:	RIN: 9.20

Fragment table for sample 1 : FKRN202334255-1A

Name	Start Time [s]	End Time [s]	Area	% of total Area
18S	40.83	43.25	18.4	19.3
28S	46.80	52.26	30.6	32.1

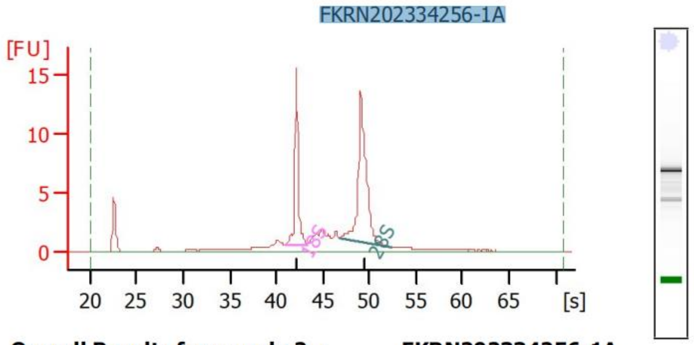

Overall Results for sample 2 : FKRN202334256-1A

RNA Area:	77.8
RNA Concentration:	146 ng/µl
rRNA Ratio [28s / 18s]:	1.7
RNA Integrity Number (RIN):	9.3 (B.02.10)
Result Flagging Color:	
Result Flagging Label:	RIN: 9.30

Fragment table for sample 2 : FKRN202334256-1A

Name	Start Time [s]	End Time [s]	Area	% of total Area
18S	40.89	43.30	15.0	19.3
28S	46.85	52.24	25.8	33.2

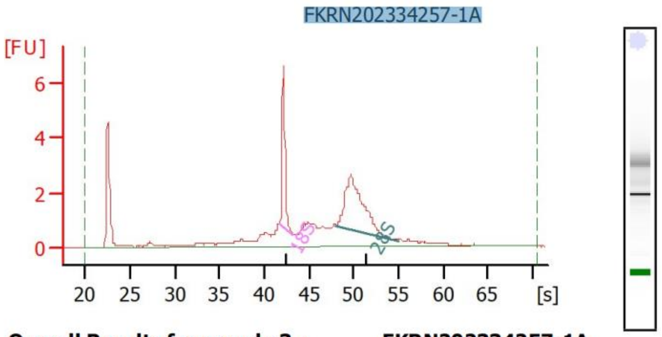

Overall Results for sample 3 : FKRN202334257-1A

RNA Area:	41.3
RNA Concentration:	77 ng/µl
rRNA Ratio [28s / 18s]:	1.6
RNA Integrity Number (RIN):	8.4 (B.02.10)
Result Flagging Color:	
Result Flagging Label:	RIN: 8.40

Fragment table for sample 3 : FKRN202334257-1A

Name	Start Time [s]	End Time [s]	Area	% of total Area
18S	41.73	43.26	5.6	13.5
28S	48.07	55.02	9.1	22.0

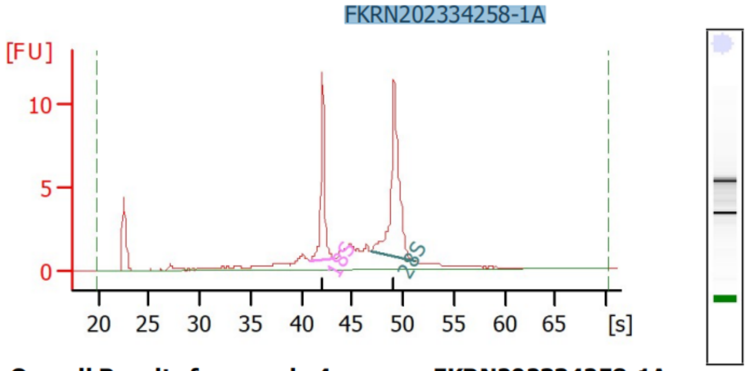

Overall Results for sample 4 : FKRN202334258-1A

RNA Area:	66.9
RNA Concentration:	125 ng/µl
rRNA Ratio [28s / 18s]:	1.8
RNA Integrity Number (RIN):	8.9 (B.02.10)
Result Flagging Color:	
Result Flagging Label:	RIN: 8.90

Fragment table for sample 4 : FKRN202334258-1A

Name	Start Time [s]	End Time [s]	Area	% of total Area
18S	40.83	43.32	11.0	16.5
28S	46.83	51.11	19.9	29.7

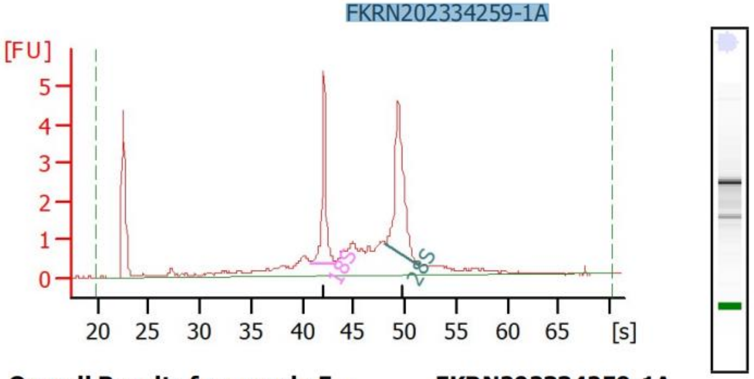

Overall Results for sample 5 : FKRN202334259-1A

RNA Area:	35.3
RNA Concentration:	66 ng/µl
rRNA Ratio [28s / 18s]:	1.5
RNA Integrity Number (RIN):	8.6 (B.02.10)
Result Flagging Color:	
Result Flagging Label:	RIN: 8.60

Fragment table for sample 5 : FKRN202334259-1A

Name	Start Time [s]	End Time [s]	Area	% of total Area
18S	40.77	43.27	5.1	14.3
28S	48.11	51.36	7.8	22.1

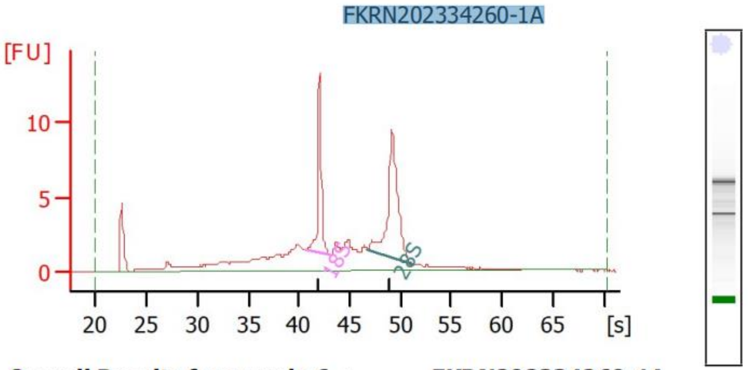

Overall Results for sample 6 : FKRN202334260-1A

RNA Area:	96.2
RNA Concentration:	180 ng/µl
rRNA Ratio [28s / 18s]:	1.6
RNA Integrity Number (RIN):	7.5 (B.02.10)
Result Flagging Color:	
Result Flagging Label:	RIN: 7.50

Fragment table for sample 6 : FKRN202334260-1A

Name	Start Time [s]	End Time [s]	Area	% of total Area
18S	40.66	43.06	12.2	12.7
28S	46.79	51.38	19.8	20.6

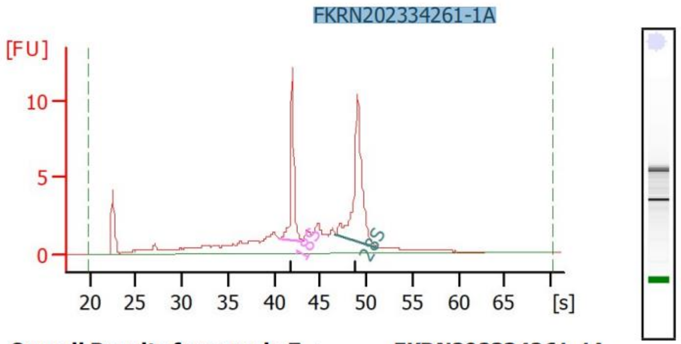

Overall Results for sample 7 : FKRN202334261-1A

RNA Area:	84.3
RNA Concentration:	158 ng/µl
rRNA Ratio [28s / 18s]:	1.7
RNA Integrity Number (RIN):	7.8 (B.02.10)
Result Flagging Color:	
Result Flagging Label:	RIN: 7.80

Fragment table for sample 7 : FKRN202334261-1A

Name	Start Time [s]	End Time [s]	Area	% of total Area
18S	40.62	43.01	11.3	13.4
28S	46.68	51.21	18.8	22.3

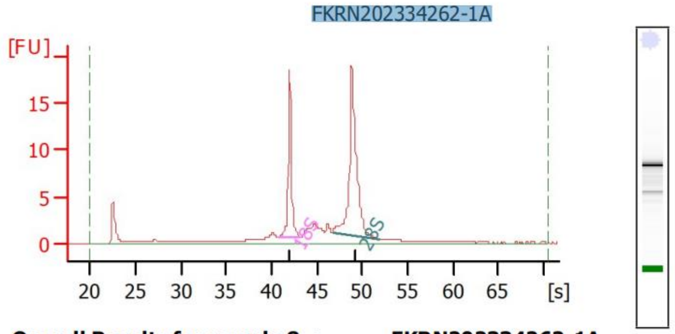

Overall Results for sample 8 : FKRN202334262-1A

RNA Area:	97.6
RNA Concentration:	183 ng/µl
rRNA Ratio [28s / 18s]:	1.8
RNA Integrity Number (RIN):	9.2 (B.02.10)
Result Flagging Color:	
Result Flagging Label:	RIN: 9.20

Fragment table for sample 8 : FKRN202334262-1A

Name	Start Time [s]	End Time [s]	Area	% of total Area
18S	40.76	43.16	17.8	18.2
28S	46.69	51.85	32.8	33.6

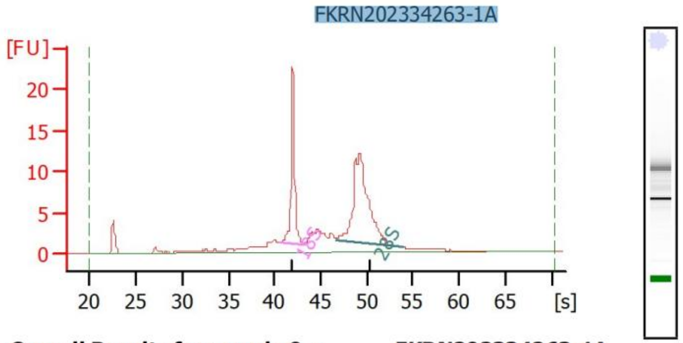

Overall Results for sample 9 : FKRN202334263-1A

RNA Area:	127.0
RNA Concentration:	238 ng/µl
rRNA Ratio [28s / 18s]:	1.8
RNA Integrity Number (RIN):	9.2 (B.02.10)
Result Flagging Color:	
Result Flagging Label:	RIN: 9.20

Fragment table for sample 9 : FKRN202334263-1A

Name	Start Time [s]	End Time [s]	Area	% of total Area
18S	40.66	43.11	23.4	18.4
28S	46.68	53.93	41.4	32.6

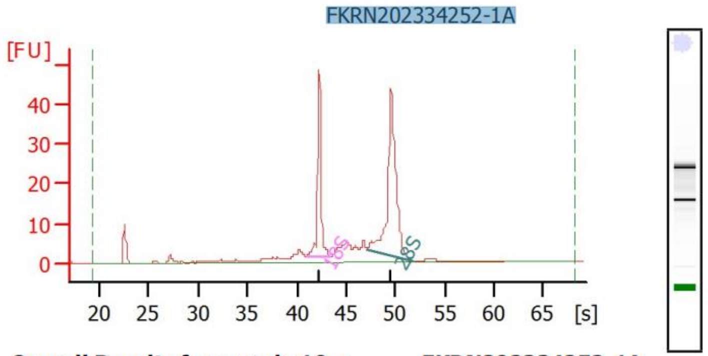

Overall Results for sample 10 : FKRN202334252-1A

RNA Area:	221.6
RNA Concentration:	175 ng/µl
rRNA Ratio [28s / 18s]:	1.9
RNA Integrity Number (RIN):	9.3 (B.02.08)
Result Flagging Color:	
Result Flagging Label:	RIN: 9.30

Fragment table for sample 10 : FKRN202334252-1A

Name	Start Time [s]	End Time [s]	Area	% of total Area
18S	40.99	43.42	42.3	19.1
28S	47.13	51.73	81.3	36.7

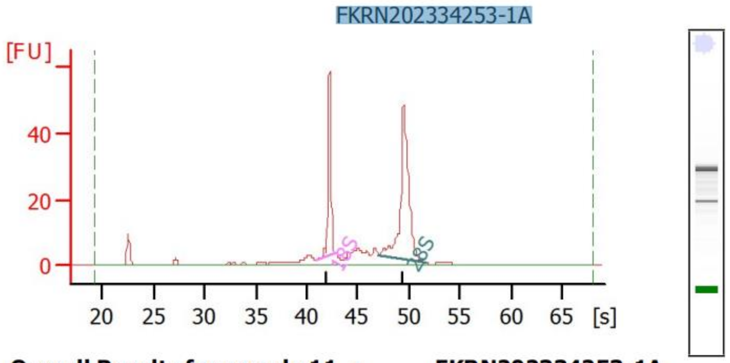

Overall Results for sample 11 : FKRN202334253-1A

RNA Area:	226.5
RNA Concentration:	179 ng/µl
rRNA Ratio [28s / 18s]:	2.0
RNA Integrity Number (RIN):	9.4 (B.02.08)
Result Flagging Color:	
Result Flagging Label:	RIN: 9.40

Fragment table for sample 11 : FKRN202334253-1A

Name	Start Time [s]	End Time [s]	Area	% of total Area
18S	41.00	42.78	43.9	19.4
28S	47.12	51.61	89.0	39.3

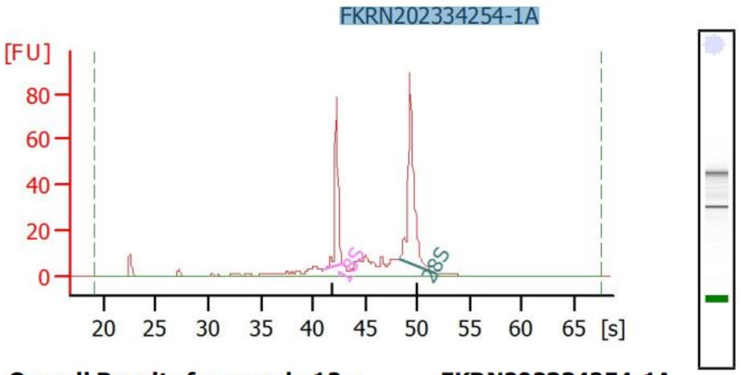

Overall Results for sample 12 : FKRN202334254-1A

RNA Area:	331.5
RNA Concentration:	261 ng/µl
rRNA Ratio [28s / 18s]:	1.7
RNA Integrity Number (RIN):	9.2 (B.02.08)
Result Flagging Color:	
Result Flagging Label:	RIN: 9.20

Fragment table for sample 12 : FKRN202334254-1A

Name	Start Time [s]	End Time [s]	Area	% of total Area
18S	41.08	42.84	62.9	19.0
28S	48.28	51.76	105.7	31.9
